# A Randomized Clinical Trial Comparing Breakfast and Bedtime Administration of Insulin Glargine in Children and Adolescents with Type 1 Diabetes

**DOI:** 10.4008/jcrpe.v1i1.10

**Published:** 2008-08-03

**Authors:** Damla Gökşen Şimşek, Başak Yıldız, Gülgün Asar, Şükran Darcan

**Affiliations:** 1 Ege University, Faculty of Medicine, Pediatric Endocrinology and Metabolism Unit, İzmir, Turkey; +90−232 388 63 66+90−232 388 63 66damla.goksen@ege.edu.trEge Üniversitesi Tıp Fakültesi Pediatrik Endokrinoloji ve Metabolizma BD 35100 Bornova−İzmir−Turkey

**Keywords:** Glargine insulin

## Abstract

**Background**: Insulin glargine provides effective glycemic control when administered at bedtime in adults.

**Objective**: This study aims to investigate whether insulin glargine is equally effective if administered in the morning or at bedtime in combination with preprandial anologue insulin.

**Methods**: Twenty−eight patients that have been treated with an intensified insulin regimen for at least one year were randomized to insulin glargine injection at breakfast (06:00−09:00) (12 patients) or bedtime (21:00−24:00) (16 patients), plus meal−time anologue insulin in the two groups. Glucose data from each day were analyzed at four different times: between 9:00 and 21:00 (t1), between 21:00 and 24:00 (t2), between 24:00 and 04:00 (t3),04:00 and 09:00 (t4) by the Minimed continuous glucose monitoring system.

**Results**: Baseline characteristics were similar in the two groups. The sensor values were lower before breakfast in the bedtime group (180.5 ± 49.0 vs 223.8 ± 47.3 mg/dl, p=0.03). There were 13.7 events.patient ^−1^.day^−1^ in the bedtime group and 6.9 events.patient ^−1^.day^−1^ in the breakfast group in which glucose levels fell below 60 mg/dl (p=0.3). There were 121.6 events.patient ^−1^.day^−1^ in the bedtime group and 162.4 events.patient ^−1^.day^−1^ in the breakfast group in which glucose levels exceeded 180 mg/dl (p=0.05). Nighttime hypoglycemia only reached to a statistical significance between the two groups between 24:00 and 04:00. There were no significant correlations between the duration of nocturnal hypoglycemia, age, duration of diabetes, gender and HbA1c levels.

**Conclusion**: Breakfast group is hyperglycemic during the day and hyperglycemia starts in the morning at 04:00. There is no significant difference in the frequency or duration of hypo/hyper glycemia during the day and night irrespective of the timing of glargine injection except pre−breakfast levels are significantly better in the bedtime group and hypoglycemia occurs between midnight and 04:00 in the bedtime group.

**Conflict of interest:**None declared.

## INTRODUCTION

The time action profile of insulin glargine has been demonstrated to provide a continuous, smooth supply of insulin with no pronounced peak over a 24 h period. This unique action profile enables individual tailoring of the timing of basal insulin action.(1) In a combination regimen with prandial insulin, insulin glargine provides equivalent levels of glycemic control and reduces the frequency of hypoglycemia when compared to NPH insulin.(2, 3, 4) There is only one adult and one pediatric study comparing the regimen of intensive basal insulin supplementation with once daily glargine administration at breakfast versus bedtime.(5, 6)

The aim of this study was to investigate whether insulin glargine is equally effective if administered in the morning or at bedtime in combination with preprandial insulin analogue on glycemic control and nocturnal hypo/hyperglycemia, based on blood glucose values from 24 hour glucose monitorization system (CGMS) (Medtronic Minimed [Northridge CA]) on the 12^th^ week when the metabolic control had been stabilized.

## MATERIALS AND METHODS

**Patient Population**

A total of 28 patients with type 1 diabetes were recruited for this study during routine outpatient visits. The mean age of the subjects were 12.2±4.3 years, (4.8−20.8 years). Patients had a mean duration of diabetes of 4.8±3.5 years and a mean HbA1c of 8.7±1.9%.

**Study Protocol**

In this open label, randomized study, 28 patients (14 females, 14 males) that have been treated with an intensified insulin regimen (neutral protamine Hagedorn (NPH)+Regular insulin (R)/or NPH+Lispro/ aspart) for at least one year were randomized to insulin glargine (Lantus Aventis Pharma) injection at breakfast−time (06:00−09:00) (6 females, 6 males), or at bedtime (21:00−24:00) (8 females, 8 males), each plus meal−time rapid−acting insulin analogue (16 bedtime insulin, 12 breakfast insulin) after a 2 weeks screening phase where patients continued their usual insulin regimen and were encouraged to strive for optimal glycemic control. Total daily basal insulin glargine dose was calculated as 45% of the total dose. Basal insulin was titrated by the patient based on home self monitored glucose measurements (SMBG) according to the predefined premeal blood glucose of 80−120 mg/dL. Short acting insulin anologue was titrated individually as necessary, aiming to reach a postprandial glucose of no more than 140 mg/dL.

CGMS was performed at the end of the 12th week of insulin glargine treatment. At that time the patients came in for an outpatient visit for sensor insertion and they were instructed to keep detailed logs during this period and record events, insulin dosage and exercise and were trained how to use CGMS. CGMS sensor replacement was done by a certified diabetes nurse. Calibration of the sensor was accomplished by the protocol established as outlined in the Minimed CGMS manual. At the completion of the CGMS period the system was returned and the data were downloaded and converted into glucose levels using Minimed Solution software version 3.0. Logbooks were also analyzed to determine finger stick blood glucose levels.

Weight and height were measured by standard methods and body mass index (BMI) was calculated accordingly (kg/m2) and expressed as standard deviation score (SDS) according to Cole et al.(7)

**Data Analyses**

Glucose data from each day were analyzed at four different time periods: between 9:00 and 21:00 (t1), between 21:00 and 24:00 (t2), between 24:00 and 04:00 (t3), 04:00 and 09:00 (t4). 

Glycemic excursions were defined as hypoglycemia <60 mg/dL and hyperglycemia as any sensor value >180 mg/dL.

The SMBG values were entered into the CGMS monitor to obtain correlation coefficients between the SMBG and the CGMS values. We analyzed the data if 288 sensor values per 24 h were available. Average glucose concentration per 24 h, average glucose concentration during the time periods given above, as well as number of excursions, time and area under the glucose curve (AUC) above 180 mg/dL and below 60 mg/dL were calculated from the CGMS data. Additionally, average glucose concentration per 24 hour was calculated from the results of the standardized self monitoring of blood glucose performed by the patients. The following cutoff criteria for optimal accuracy were adhered to: the correlation coefficient between the sensor and the meter readings was above the cutoff point of 0.79 and a mean absolute difference (MAD) of sensor and meter readings was below 28%.

Glycated hemoglobin (HbA_1c_) was measured by high performance liquid chromatography at onset and at 12^th^ week of the study.

SPSS (version 11.0) statistical software was used for analyses. All values are presented as mean±SD. Nonparametric tests were used for comparison between the mean values. P values of less than 0.05 were considered significant.

All patients and families were informed about the study and consents were obtained.

## RESULTS

Baseline characteristics of the patients were similar in the two groups (Table 1). All the patients completed 12 weeks of insulin glargine therapy. During home use 26 of the 28 patients used CGMS for >60 h, which included three overnight profiles. In the two children the sensor was disconnected. In one of the children the first sensor had to be replaced in the first day. The system was well tolerated by all subjects.

An insignificant reduction of mean HbA_1c_ from baseline to end point (end of 12 weeks) occurred in both of the groups (Table 2).

**Overall glycemic control**

The overall mean of the SMBG levels obtained by the subjects before meals during the three days of sensor use was 179.3±35.5 and 197.2±29.6 mg/dL in the bedtime and breakfast group respectively. The average of the sensor readings obtained during the day and night over the entire 3 day period were similar (187±43.4 vs 204.0±23.4 mg/dL respectively) (Table 3).

There were 13.7 events.patient^−1^.day^−1^ in the bedtime group and 6.9 events.patient^−1^.day^−1^ in the breakfast group in which sensor glucose levels fell below 60 mg/dL all through the day (p=0.3).

There were 121.6 events.patient^−1^.day^−1^ in the bedtime group and 162.4 events. patient^−1^.day^−1^ in the breakfast group in which sensor glucose levels exceeded the target of 180 mg/dL throughout the day (p=0.05).

**Daytime glycemic control (09:00 to 21:00)**

The pre and post meal sensor values tended to be lower before breakfast in the bedtime group (Table 4). Peak postprandial values were near our target range (180 mg/dL).

There were 8.5 events.patient^−1^.day^−1^ in the bedtime group and 0.9 events.patient^−1^. day^−1^ in the breakfast group in which sensor glucose levels fell below 60 mg/dL between 09:00 and 21:00 (p=0.3). Sensor glucose levels were below 60 mg/dL for a mean of 127.5±354.6 and 13.8±23.5 min/daytime for the bedtime and breakfast group respectively (p=0.2).

There were 54.2 events.patient^−1^.day^−1^ in the bedtime group and 77.5 events.patient^−1^.day^−1^ in the breakfast group in which sensor glucose levels exceeded the target of 180 mg/dl between 09:00 and 21:00 (p=0.05). Sensor glucose levels were >180 mg/dl for a mean of 83.3±149.4 and 90.8±65.9 min/daytime for the bedtime and breakfast group respectively (p=0.9).

**Nighttime (21:00 to 09:00) glycemic control**

When glucose levels from all nocturnal profiles were analyzed, sensor glucose levels were below 60 mg/dl for a mean of 28.4±49.8 and 30.2±76.0 min/night for the bedtime and breakfast group respectively (p=0.9).

Nighttime hypoglycemia did not reach to a statistical significance between the two groups except in the time interval 2 (t2). The frequency of hypoglycemia during t2 was 2.4 events.patient^−1^.night^−1^ vs 0.0 (p=0.005). The frequency of hyperglycemia did not reach to a significant difference between the two groups in none of the time periods during night.

The duration of hyper− and hypoglycemic periods throughout the day are given in Table 5.

43.8% of the bedtime group had nighttime hypoglycemia whereas 33.3% of the breakfast group had nocturnal hypoglycemia. There were no significant correlations between the duration of nocturnal hypoglycemia and patient age, duration of diabetes and HbA_1c_ levels. Similarly gender did not significantly affect nocturnal hypoglycemia duration.

**Table 1:**
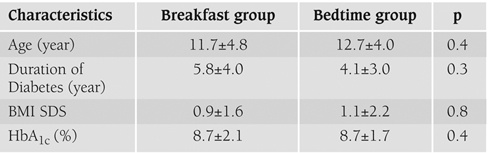
Characteristics of the two groups

**Table 2:**
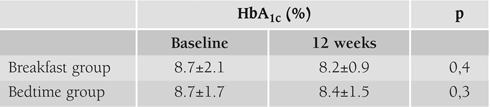
HbA_1c_ at baseline and at 12 weeks in two groups

**Table 3:**
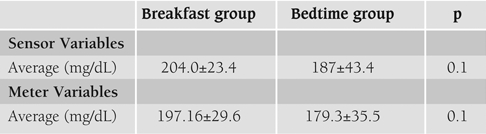
Sensor and meter variables throughout 3 days

**Table 4:**
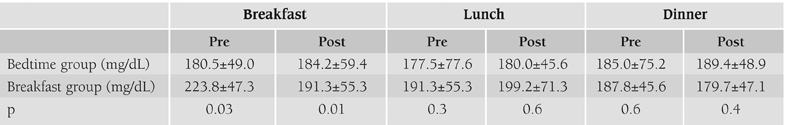
Pre and post meal sensor values

**Table 5:**
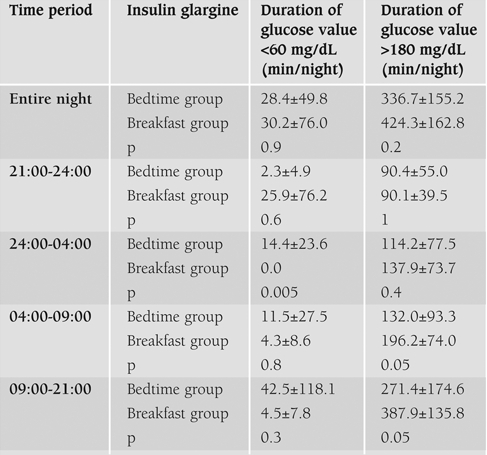
Duration of hyper and hypoglycemic periods

## DISCUSSION

The aim of this study was to investigate whether insulin glargine is equally effective if administered in the morning or at bedtime in combination with preprandial insulin anologue on glycemic control and nocturnal hypo/hyperglycemia; based on blood glucose values from 24 hour glucose monitorization system. This data shows that there was no significant difference in the frequency or duration of hypo/hyperglycemia during the day and night irrespective of the timing of glargine injection. Breakfast glargine protected only from hypoglycemia occurring in between 24:00 and 04:00. Breakfast group was hyperglycemic during the day and hyperglycemia started in the morning at 04:00.

Minor reductions in HbA_1c_ after 12 weeks of treatment were noted in both of the groups as shown by Hamann et al(5) in type 1 diabetics aged 18−68 years at the end of 24 weeks. Karaguzel et al(6) had shown significant reduction in HbA_1c_ at the end of six months of therapy with insulin glargine only in the breakfast group. In poorly controlled children and adolescents insulin glargine at lunch time at the end of six months of therapy showed a statistically significant drop in HbA_1c_.(8) In twenty−six adolescents who have received 4 months of bedtime insulin glargine with pre−meal insulin lispro and 4 months of bedtime NPH with pre−meal regular insulin fasting glucose levels were lower on the glargine regimen, but the hemoglobin A1c level was not statistically different (8.7 vs. 9.1%).(9)

Studies have shown lower rate of nocturnal hypoglycemic events in patients with type 1 diabetes on basal bolus regimen involving insulin glargine as the basal substitution given at bedtime compared with NPH insulin.(10, 11)

Hypoglycemic events/patient/day between the two groups were not different. A slightly higher rate of hypoglycemic events was found in the bedtime group during the daytime. Although it has been stated that the time action profile of glargine can decrease the risk of nocturnal hypoglycemia and should be given preferentially at bedtime(5) there was a difference in the incidence of nocturnal hypoglycemia in time interval t3 between the bedtime and breakfast group (2.4 events.patient^−1^.night^−1^ vs. 0). Hamann et al(5) reported that the incidences of total symptomatic and severe hypoglycemia did not differ between dinner, bedtime and breakfast glargine insulin groups however nocturnal hypoglycemia occurred in significantly fewer patients in the breakfast group compared with the dinner and bedtime groups. In the study of Karagüzel et al(6) the administration of once daily insulin glargine at breakfast or bedtime was associated with a decreased risk of all type of hypoglycemic events. An insignificant increase of nocturnal hypoglycemia during the time interval t1 in the breakfast group could be due to incorrect adjustments of premeal insulin during dinner since this time interval corresponds to post meal.

The results of this study demonstrate that insulin glargine in combination with insulin lispro/aspart is equally effective on glycemic control whether it is injected once daily before breakfast or at bedtime in type 1 diabetic adolescents and children. There is no significant difference in the frequency or duration of hypo− and hyperglycemia during the day and night irrespective of the timing of glargine injection except pre breakfast levels are significantly better in the bedtime group and hypoglycemia occurs in between midnight and 04:00 in the bedtime group.
